# Supplementary figures and images for: The differential expression of MAGI2 in glomerulopathies and its application as a molecular discriminator of podocytopathies
Source: J Transl Med. 2025 Jun 25;23:701. doi: 10.1186/s12967-025-06696-9 (PMC12199530; doi:10.1186/s12967-025-06696-9)

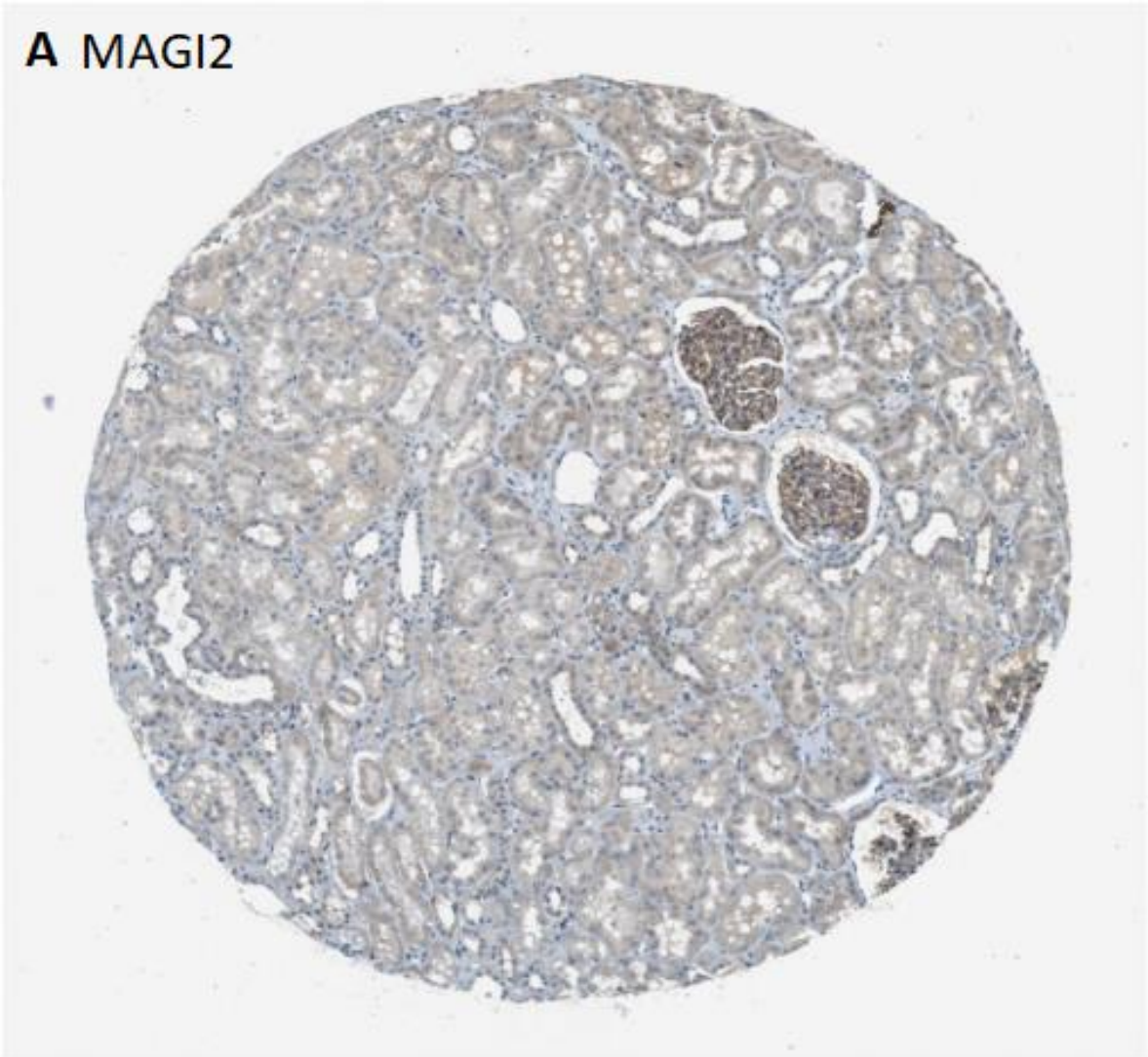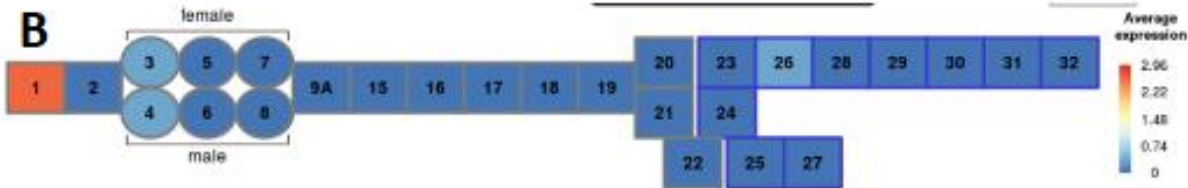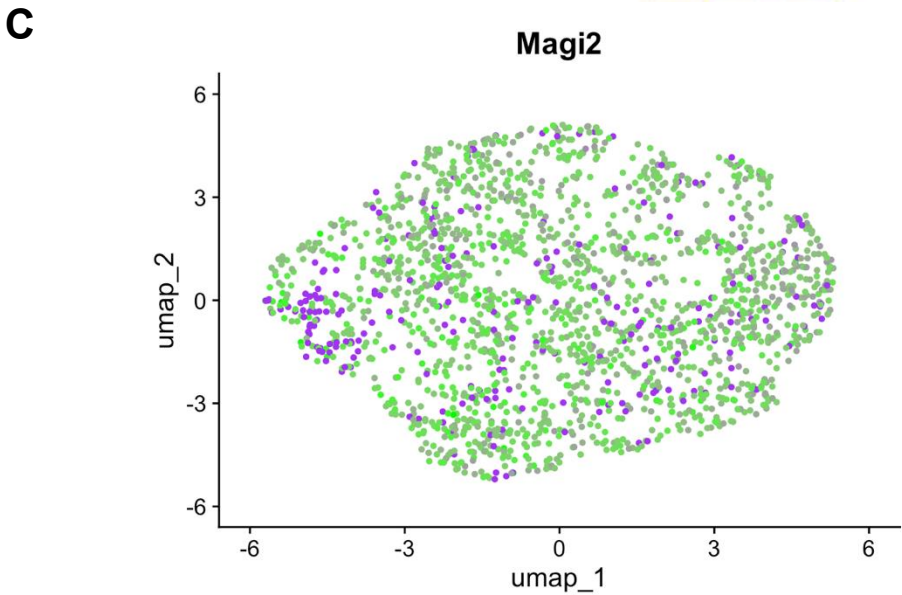

Suppl. Fig. 2

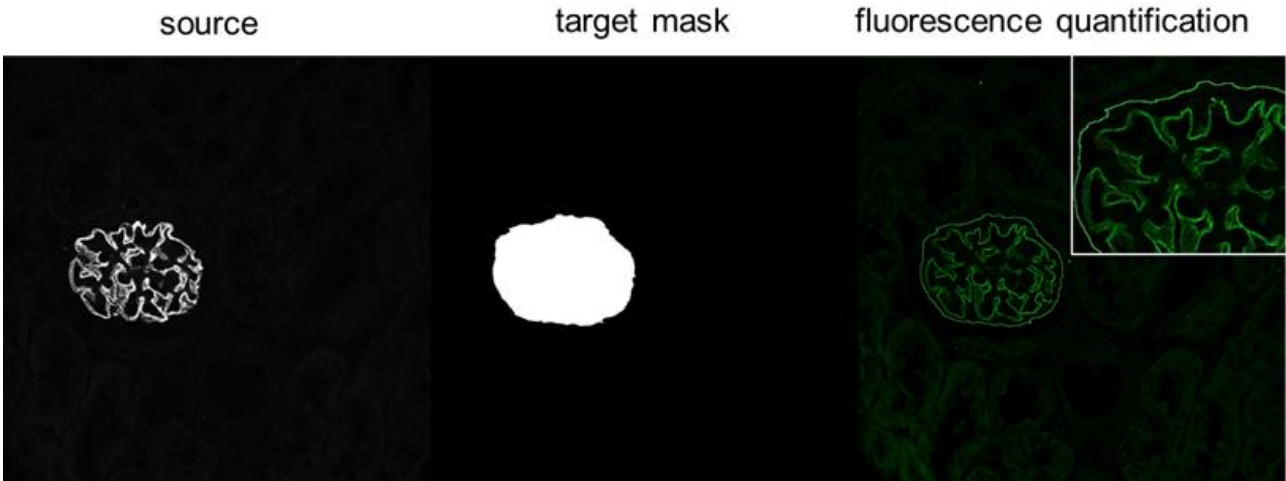

Supplement: Supplementary file 1 — Supplementary Material 1. [file 12967_2025_6696_MOESM1_ESM.pdf]
